# Use of ketamine for treatment resistant depression: updated review of literature and practical applications to a community ketamine program in Edmonton, Alberta, Canada

**DOI:** 10.3389/fpsyt.2023.1283733

**Published:** 2024-01-08

**Authors:** Carson Chrenek, Bryan Duong, Atul Khullar, Chris McRee, Rejish Thomas, Jennifer Swainson

**Affiliations:** ^1^Department of Psychiatry, Misericordia Community Hospital, University of Alberta, Edmonton, AB, Canada; ^2^Department of Psychiatry, University of Alberta Hospital, University of Alberta, Edmonton, AB, Canada; ^3^Department of Psychiatry, Grey Nuns Community Hospital, University of Alberta, Edmonton, AB, Canada; ^4^Grey Nuns Community Hospital, Edmonton, AB, Canada

**Keywords:** ketamine, non-intravenous ketamine, maintenance ketamine, community ketamine use, depression, treatment-resistant depression

## Abstract

**Background:**

Though intravenous (IV) ketamine and intranasal (IN) esketamine are noted to be efficacious for treatment-resistant depression (TRD), access to each of these treatments within healthcare systems is limited due to cost, availability, and/or monitoring requirements. IV ketamine has been offered at two public hospital sites in Edmonton, Canada since 2015. Since then, demand for maintenance ketamine treatments has grown. This has required creative solutions for safe, accessible, evidence-based patient care.

**Objectives:**

Aims of this paper are twofold. First, we will provide a synthesis of current knowledge with regards to the clinical use of ketamine for TRD. Consideration will be given regarding; off-label racemic ketamine uses versus FDA-approved intranasal esketamine, populations treated, inclusion/exclusion criteria, dosing, assessing clinical response, concomitant medications, and tolerability/safety. Second, this paper will describe our experience as a community case study in applying evidence-based treatment. We will describe application of the literature review to our clinical programming, and in particular focus on cost-effective maintenance treatments, long-term safety concerns, routes of ketamine administration other than via intravenous, and cautious prescribing of ketamine outside of clinically monitored settings.

**Methodology:**

We conducted a literature review of the on the use of ketamine for TRD up to June 30, 2023. Key findings are reviewed, and we describe their application to our ketamine program.

**Conclusion:**

Evidence for the use of ketamine in resistant depression has grown in recent years, with evolving data to support and direct its clinical use. There is an increasing body of evidence to guide judicious use of ketamine in various clinical circumstances, for a population of patients with a high burden of suffering and morbidity. While large-scale, randomized controlled trials, comparative studies, and longer-term treatment outcomes is lacking, this community case study illustrates that currently available evidence can be applied to real-world clinical settings with complex patients. As cost is often a significant barrier to accessing initial and/or maintenance IV or esketamine treatments, public ketamine programs may incorporate SL or IN ketamine to support a sustainable and accessible treatment model. Three of such models are described.

## 1 Introduction

Treatment-resistant depression (TRD) has been estimated to affect 30–55% of individuals with Major Depressive Disorder (MDD) (1). Though definitions of TRD vary in the literature, one accepted definition is a failure to respond to two (or more) first-line antidepressant agents with adequate dose and duration, and it has been noted that nearly a third of individuals with MDD do not remit with the first or second treatment step (2). Ketamine is an NMDA-antagonist that has demonstrated rapid efficacy as a novel antidepressant in numerous systematic reviews and meta-analyses (3–7). Note that for the purposes of this paper, discussion will center around the pharmacologic use of ketamine for depression, and exclude any discussion of ketamine assisted psychotherapy.

In 2015, due to an unmet need, the Gray Nuns and Misericordia Hospitals (Covenant Health, Edmonton, Canada) began offering limited, publicly funded intravenous (IV) ketamine treatments as a novel treatment option for selected patients with severe treatment resistant unipolar or bipolar depression. Due to its limited evidence at that time, our programs initially treated only patients who had exhausted all other treatment options. Early patients in our program were considered to have ultra-resistant depression (URD), with 90% of patients failing to respond to electroconvulsive therapy (ECT) and an average of 8.1 prior antidepressant trials (8). This represents a greater level of treatment resistance than the typical patients included in IV ketamine studies. Based on chart review of these first 50 patients treated in our program, 50% had unipolar depression, 40% had bipolar depression, and 10% were unspecified. 44% responded within 8 IV treatments of ketamine and 16% remitted. Controlled studies have since corroborated that reduced (but still meaningful) efficacy is still likely to occur in patients with similarly high degrees of treatment resistance (9–11).

Data on the safety and efficacy of ketamine for depression has subsequently grown substantially. In Canada, due to the largest body of evidence available, IV racemic ketamine has been acknowledged as a 3rd line treatment for both bipolar depression and adults with unipolar TRD (12, 13). While the majority of studies have involved unipolar (or mixed unipolar/bipolar) TRD, a recent review confirmed similar efficacy and tolerability in studies with exclusively bipolar depression (14). Similarly, intranasal (IN) esketamine has been approved in Canada and the United States for TRD with the above definition (15). As a result, our inclusion criteria were broadened in 2020 to include “less” treatment resistant individuals. These local protocols served as the basis for a broader provincial IV ketamine protocol for depression to be used in Alberta, Canada (16). Due to high cost and lack of public coverage for most, IN esketamine, though indicated for TRD is not used by our program. As such, this review focuses on the use of racemic ketamine for depression as has been used in our programs.

With burgeoning evidence and increasing mass media popularity, the demand for ketamine treatment has risen, and there has been a rapid increase in the number of clinics and hospital sites that are using ketamine for TRD (17). It would follow that these programs have addressed, or will need to address clinical issues that arise, such as issues around maintenance treatments and ways to increase access as IV ketamine programs become saturated. As the literature is rapidly growing, our group sought to review current literature to ensure best practices in our program. We do not intend to provide an exhaustive review of the literature, although the findings of several recent systematic reviews and meta-analysis will be described in this paper.

This document will review key questions that we have evaluated, and how it has been applied to our program. We will also discuss the models of non-parenteral ketamine use we have considered as options to increase overall access to ketamine treatment for depression within public healthcare systems.

## 2 Methods

The Canadian Network for Mood and Anxiety Treatments (CANMAT) 2020 Ketamine Task force update was considered a comprehensive systematic review on ketamine for depression as a baseline. As this paper’s literature search covered to Jan 31, 2020, we conducted a review of the literature from January 1, 2020 – June 30, 2023. This search was done via OVID search platform, MEDLINE database. The keyword terms ‘ketamine’ or ‘esketamine’ were used; combined with ‘depression’ or ‘bipolar” or ‘TRD’ or ‘treatment resistant depression’. Age groups were selected for 19 years and older, with studies limited to humans. Case reports, clinical trials, comparative studies, practice guidelines, meta-analysis, multicentre studies, observational studies, randomized controlled trials, reviews, and systematic reviews were considered. Reference lists of papers were also scanned for additionally relevant items. Papers were discussed among authors, and key items were brought to the interdisciplinary ketamine team for further review. Other notable studies outside of these parameters or suggested by peer reviewers between the end of our literature review and final manuscript acceptance were considered and added when felt to add value to the manuscript. Literature has been synthesized in the following discussion along with the authors’ suggested applications to clinical practice.

## 3 Clinical considerations

### 3.1 What are our considerations in choosing between IV ketamine or in esketamine?

IN esketamine (SPRAVATO) was approved by Health Canada for TRD in 2020, while off-label, IV ketamine was previously acknowledged as an effective adjunct for TRD. While there is more robust clinical trial data for IN esketamine, its high cost frequently precludes its use. In terms of efficacy, head-to-head RCTs between IV ketamine and IN esketamine are lacking, but metanalyses of observational studies have compared efficacy of both treatments. One meta-analysis showed no difference in efficacy up to 1 month (18). However, two recent studies suggest that while each had similar rates of response/remission, IV ketamine required fewer treatments to achieve this outcome (19, 20).

Regardless, as racemic IV ketamine is not prohibitive in cost to the patients in our program (it is covered by public healthcare), it is the standard of treatment we use and will be the focus of discussion.

### 3.2 What population should be treated with IV ketamine?

Our protocol currently applies only to individuals with TRD (unipolar or bipolar), defined as failure to respond to two or more trials of appropriate pharmacotherapy. Our program treats adult patients ages 18 and over, including adults over 65, as there is early data for efficacy and safety with ketamine (21, 22) in older adults, and even more data with esketamine (23). Though not part of our patient population, one randomized control trial (RCT) in adolescents with TRD has also had favorable results (24).

Our population of adult patients with TRD is largely heterogeneous in terms of comorbidities, illness severity/duration, and levels of treatment resistance. More treatment-resistant patients have been reported as less likely to fully remit, but not less likely to respond to treatment (17). A recent study demonstrated that clinical features including severe anhedonia, anxious distress, mixed symptoms and/or bipolarity were more highly associated with response/remission (25). Efficacy of IV ketamine in individuals has been reported in two meta-analyses as either slightly inferior, or not different from ECT (26, 27). Evidence for functional improvement with ketamine treatment is lacking, but data supports the general notion that psychosocial functioning improves (28). Qualitatively, we have seen numerous cases of resistant patients who respond to treatment in a functionally meaningful way that improves quality of life and merits consideration for ongoing treatment. While there is preliminary evidence for use of ketamine in obsessive-compulsive disorder, social anxiety, post-traumatic stress disorder, psychosis, and comorbid substance use disorders (29), none were considered robust enough for inclusion in a regular protocol.

### 3.3 What is the inclusion/exclusion criteria for IV ketamine?

The presence of psychiatric comorbidities (including borderline personality disorder) does not significantly affect treatment outcomes or efficacy in a meaningful way (30, 31). Exclusion criteria include a primary psychotic disorder, uncontrolled hypertension, central aneurysmal disease, significant valvular disease, recent cardiovascular event (within 6 weeks), and class 3 heart failure (New York Heart Association) as per CANMAT recommendations (32).

Pregnancy and breastfeeding are considered contraindications to IV ketamine. While brief exposure to ketamine in the context of general anesthesia is unlikely to have negative effects, ketamine is known to cross the placenta (33) and exposure to repeated doses has not been studied. Animal models also suggest potential for adverse events with exposure to fetuses and infants (34).

Exclusion criteria have been made relative, rather than absolute, in keeping with longtime considerations to determine eligibility for ECT. Medical and/or second consultation with a psychiatrist is sought when appropriate to aid in assessment of risk/benefit. Decisions are made on a case-by-case basis.

### 3.4 Dosing regimens for IV ketamine

Meta-analysis of 79 studies (2,665 patients) reported variable but significant and conclusive efficacy for both response and remission rates with single and repeated ketamine dosing (35). With repeated treatments, ketamine’s antidepressant effect was maintained, and appears to offer greater efficacy and more prolonged benefit compared to single infusion (36).

The standard acute course in our program is 8 treatments, typically administered two times weekly. Though three-times weekly treatments are no more effective than twice-weekly (37), some patients in our program may receive 1 or 3 treatments in a week depending on scheduling availability.

A dose of 0.5 mg/kg has been previously recommended (12), as lower doses have not been found to be as effective (38). A higher single dose of 1.0 mg/kg was found similarly safe, but not more effective than 0.5 mg/kg. There was a trend toward a longer duration of response at the higher dose, however.

**Figure 1:**
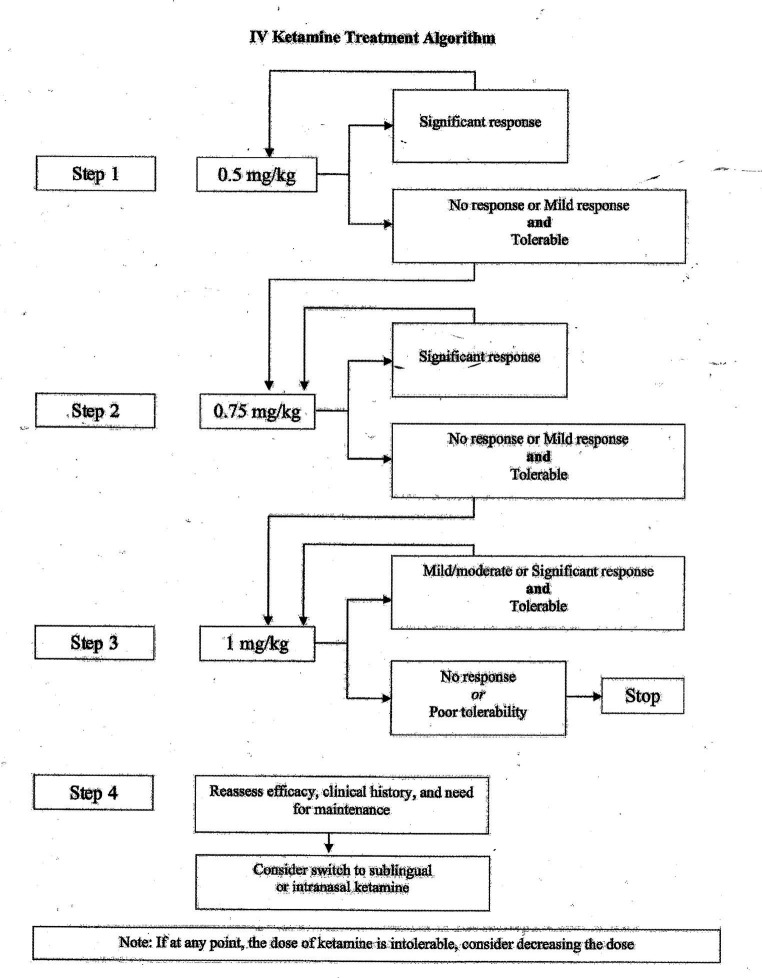
Suggested treatment algorithm for IV ketamine prescribing. If any point, the dose of ketamine is intolerable, consider decreasing the dose.

The starting dose for IV ketamine in our program is 0.5 mg/kg, infused over 40 min. Given the safety data for higher doses, and data above that suggested a longer duration of response, we began increasing doses to 0.75 mg-1.0 mg/kg for patients who were tolerating infusions but had little or no response to 0.5 mg/kg. In our clinical experience, we have observed several patients who do not respond until the dose is increased, and data looking at superiority of 1.0 mg/kg versus 0.5 mg/kg was based only on a single infusion. Based on this, combined with clinical experience, Figure 1 highlights a suggested treatment algorithm. In cases where a patient only begins to respond to higher doses late in the course of 8 treatments, it is left to clinical discretion to consider extending the acute course.

### 3.5 How should clinical response be assessed?

A lack of dissociation is not correlated with reduced antidepressant response and should not be a factor in dosing decisions (39). The antidepressant response should eventually extend well beyond the treatment day, particularly after 5–8 treatments. If positive effects continue to wear off within 1–3 days, sustained antidepressant response with ketamine is unlikely and other treatment options should be considered.

It is important to ensure that there are clinical improvements in core symptoms of depression between treatments, and that the patient is not simply “liking” the dissociative effects of the treatment experience or enjoying a brief “escape” from their depression, not unlike those who abuse substances.

Along with clinical assessment, patient reported outcomes measures (PROMS) such as the Quick Inventory of Depressive Symptoms (QIDS) have been helpful to track progress. Traditional mood rating scales may not always capture rapid improvements, and another option would be the McIntyre and Rosenblat Rapid Response Scale (MARRRS), developed specifically as a tool to detect improvement with rapid acting antidepressants (40). Functional rating scales and quality of life scales may also be of benefit.

### 3.6 Which concomitant medications should be avoided?

There is mixed literature as to whether certain concomitant psychotropic medications may prevent antidepressant effects of ketamine. Though it has been suggested that benzodiazepines and other drugs acting on the Gamma-Aminobutyric Acid (GABA) systems may interfere with treatment response, a large meta-analysis found that concomitant benzodiazepine use had no overall effect (35). Though no consistent effect is found, interference has been shown with high dose benzodiazepines such as delayed response and increased likelihood of relapse (41), with delayed response also observed with benzodiazepine use in esketamine treatment. Our program recommends to reduce or discontinue benzodiazepines if possible, or to use agents with shorter half-lives and simpler metabolism. Patients are advised not to take their benzodiazepine the morning of treatment and the evening prior. Higher doses or longer courses of ketamine may be required for individuals who continue benzodiazepines.

Co-administration of a single dose of naltrexone reduced antidepressant response to ketamine in one placebo-controlled study, raising questions as to whether naltrexone should be avoided during ketamine treatment (42). Though other reports have not replicated this finding (43, 44), the uncertainty surrounding this topic suggests that the decision to continue naltrexone could be made on a case- by-case basis.

Though lamotrigine has been reported to diminish dissociative effects of ketamine, antidepressant response is still elicited, supporting the concept that dissociation is not required for pharmacologically induced antidepressant effects of ketamine (45). A systematic review concluded that there was no evidence to support a negative interaction between lamotrigine and ketamine in clinical populations (46).

It has generally been considered safe to co-administer antidepressants and mood stabilizers with ketamine, but caution has been advised with MAOIs (12). Several case reports describe safe use of esketamine and MAOIs (47), as well as ketamine with MAOIs (48). Though to be used with caution, IV ketamine has been useful as a bridging treatment during an antidepressant washout prior to starting an MAOI, and during the first few weeks of MAOI treatment.

### 3.7 What are the common acute side effects of IV ketamine?

Ketamine is a safe and well-tolerated medication when administered at antidepressant doses. Common side effects noted in literature are transient and may include psychotomimetic and dissociative experiences, blurred vision, dizziness, anxiety, irritability, headaches, nausea, tachycardia, and elevated blood pressure (6, 49, 50). Side effects peak within 30–60 min, and abate within 1–2 h. Adverse events are almost always dose-dependent (51). Patients in our program are provided this information prior to giving consent to treatment.

### 3.8 How should ketamine-induced hypertension be managed?

Ketamine is known to transiently raise blood pressure, with a mean maximum increase of 9-19 mmHg, returning to normal in 2–4 h (52). Early recommendations, based on expert consensus suggested not to proceed with IV ketamine if baseline blood pressure was over 140/90, and to pause the infusion if blood pressure exceeded 160/100 (12). A recent report suggested that up to 20% of ketamine infusions may require anti-hypertensives (51).

Conversely, transient hypertension is not treated in an emergency medicine scenario unless there are symptoms of hypertensive emergency which include crushing chest pressure, syncope, severe abdominal pain, decreased (not just altered) level of consciousness, or shortness of breath (53). Risks associated with treating asymptomatic transient hypertension have also been noted, and it could be argued that the above guidance is overzealous.

Our protocol has been updated to better align with the emergency medicine approach to management of transient hypertension. Blood pressure is measured at baseline and post-treatment. Measurements are to be taken during the infusion only if there are signs/symptoms of hypertensive emergency as noted above. Patients in our program must undergo a complete history and physical examination prior to starting a course of IV ketamine infusions. Active medical issues or untreated hypertension are considered relative exclusion criteria to be considered on an individual risk/benefit basis.

Although the aim is for chronic hypertension to be sufficiently treated prior to ketamine treatments, if baseline blood pressure is elevated on the day of treatment, the decision to proceed is made on a case-by-case basis. Otherwise healthy individuals can tolerate slight elevation in blood pressure without adverse consequences, not unlike increases seen during exercise, which is known to transiently increase systolic blood pressure to levels greater than seen with IV ketamine treatment (greater than 200 mmHg) (54). Patients with risk factors for subarachnoid hemorrhage (SAH), such as pre-existing aneurysm or arterio-venous malformation (85% of SAH clinical presentations) (55), or other medical conditions felt to be at risk with transient blood pressure elevations, would necessitate medical consultation for advice on a more cautious approach. Of note, pre-treatment with clonidine has been reported to mitigate pressor effects of ketamine without causing rebound hypertension, so this may be an option for patients where blood pressure increases may pose reason for concern (56). Use of beta blockers or calcium channel blockers has also been suggested when blood pressure is a concern (57).

### 3.9 What setting and staff are required for IV ketamine?

Though no consistent standard is in place, the Canadian Ketamine Task Force suggested that IV ketamine should be administered in a facility equipped to handle both storage of a controlled substance and ability to deal with medical emergencies. While an anesthetist need not be present to administer a subanesthetic dose, staff administering ketamine should be medical professionals with appropriate training (12). Our ketamine programs are based in acute care hospitals, which have a rapid response team available in case of an emergency. This rapid response service has never been required since the program began in 2015, with at least 10,000 infusions performed. A nurse trained in advanced airway management administers the ketamine infusion, and a physician can be reached by telephone if there are nursing concerns. Treatments are provided in the unit’s neuromodulation recovery room, or in individual patient rooms. As sensory perception is often amplified during treatment, our program aims to provide a calm environment with reduced stimuli.

Consistent nursing staff dedicated specifically to IV ketamine is beneficial, to be familiar with what to expect and how to support patients through treatments. At times, dissociative effects include tearfulness, rumination and having intrusive thoughts or memories, which may prompt nursing staff to intervene, redirect and/or support the patient. This is not considered to be a form of ketamine-assisted psychotherapy, but a supportive psychiatric nursing intervention as required. Patients are kept on the unit for approximately 90 min post-infusion, which is based on a 15-min half-life of IV ketamine, with a total time of elimination being approximately 75 min (58). If patients continue to feel dissociative effects, they are kept longer at nursing discretion. Patients require a ride home after treatment.

### 3.10 What is an approach to maintenance treatment?

Once remission is reached with a traditional antidepressant, continued treatment for a minimum of 9–12 months (or longer if it is a repeat episode or severe illness) is recommended to minimize relapse risk (59). Similarly, following a successful course of ECT, maintenance ECT reduces relapse significantly compared to pharmacotherapy alone (60). Similarly, repeated doses of IV ketamine have demonstrated efficacy in maintaining response (61). A recent systematic review included 3 RCTs, 8 open-label trials, and 30 case series with a total of 1,495 patients with bipolar or unipolar depression (62). Routes of administration varied and included IV (18 studies), IN (3), IN esketamine (5), oral (10), and intramuscular (3). There were several reports of transitioning IV ketamine patients to other dosage forms (SL, IN, oral) for ketamine maintenance treatment. The five largest (*N* = 11–94) studies of IV ketamine maintenance used dose ranges from 0.5–1.2 mg/kg. Dose frequency was variable, ranging anywhere from weekly to every 12 weeks. Important findings in this review included reports of sustained efficacy for many individuals lasting greater than 1 year, and no new safety signals with prolonged treatment.

The most robust data for maintenance ketamine comes from a large RCT of 802 patients using maintenance IN esketamine over 1 year. There was a 51% reduction in relapse with treatment-remitters, and a 70% reduction in relapse with treatment-responders, when given with conventional antidepressant compared to antidepressant plus placebo (63). The population in this report included individuals with TRD who had failed to respond to two or more antidepressants. The largest IV ketamine maintenance study to date (open-label) reported on more highly resistant individuals who had already failed to respond to ECT. Of these, 94/150 (63%) of these patients responded to IV ketamine, and with maintenance treatment of variable dose and frequency, nearly two-thirds of these highly treatment-resistant responders showed a sustained response (64).

Meta-analysis data suggests that ketamine response is less robust and of shorter duration for individuals with a higher level of treatment resistance, thus it may be these individuals for whom maintenance ketamine is a more inevitable consideration (65). Physicians in our program decide whether to offer maintenance ketamine based the degree of ketamine response, level of treatment resistance, accessibility of ongoing IV ketamine treatments, and/or patient suitability for alternate forms of ketamine use, which will be further discussed.

### 3.11 What is the long-term safety of ketamine?

There is growing but still limited data on long-term use of ketamine for depression. The previously mentioned systematic review of 1,495 patients receiving ketamine for up to 18 months did not identify safety concerns (62). A recently published survey of 6,630 patients in the United States treated with repeated or maintenance parenteral ketamine reported that discontinuation rates for adverse events was 0.7% (66). 0.5% of patients discontinued for psychological distress. There were three cases reported “bladder dysfunction,” no reports of cognitive issues, two reports of psychosis, and one report of hypomania. While the study was unable to assess causality of the adverse events, the overall incidence of these is reassuringly low. Similarly, Janssen’s esketamine clinical program reported data from a 4-year follow-up among 1,006 patients continuing to receive maintenance esketamine with no new safety signals demonstrated (67).

#### 3.11.1 Urinary effects

High dose, chronic ketamine use among ketamine abusers has been associated with ulcerative urinary cystitis and dilated common bile ducts mimicking choledochal cysts (68–71). This has not yet been reported in the literature with clinical IV ketamine treatment for depression, but we have occasionally seen patients develop transient urinary symptoms. Periodic screening for any urinary symptoms and urinalysis to screen for microscopic hematuria should be done periodically in patients using maintenance ketamine. If symptoms develop, the risk/benefit of continuing ketamine should be assessed, with urologic consultation.

#### 3.11.2 Cognition

While neurocognitive impairment has been reported with chronic ketamine abuse (72), a review of 5 IV ketamine studies with objective measurements of cognition noted either a neutral effect or an improvement in cognition, with no domains showing impairment (73). The improvement in cognition typically correlated with the degree of antidepressant response. In our program, a study of 40 patients found improved cognition over a course of 8 IV ketamine treatments as measured by a Digit Span Substitution Test (DSST) and patients perceived their overall cognition as improved when self-rated with a Perceived Deficits Questionnaire (PDQ) (74).

#### 3.11.3 Ketamine abuse/misuse

Ketamine abuse often occurs with other substance use, more confounding health variables, and consumption at significantly higher and more frequent doses than is used for depression (75, 76). Though ketamine abuse rates are substantially higher in Eastern countries (Hong Kong, Taiwan) (34), its prevalence In North America is low; estimated at 0.4% in college students over a 1 year period (77), While preclinical studies suggested a theoretical abuse potential for ketamine, two reviews of clinical literature find no suggestion for ketamine misuse or abuse when prescribed for depression (78). Real world-studies have also replicated these findings with esketamine (79, 80).

A retrospective survey of patients in our program with patients prescribed ketamine outside of clinically monitored settings did not find any indication of misuse. Drug-liking, was variable, with a number of patients indicating a dislike for the dissociative effects of ketamine. Overall risk level appeared low, but not negligible (81). Similarly, there is only one case report of drug seeking behavior and craving in a single patient treated with esketamine (82). Similar to other medications with abuse, caution in prescribing should be exercised, but its use should not be stigmatized and potentially discourage access. Suggestions for judicious use have been previously summarized by another group that included two of our coauthors and will be reviewed in following sections (83).

### 3.12 When IV ketamine is not an option, are other routes of administration possible?

Ketamine may be administered in a variety of different routes, including IV, IM, subcutaneous (SC), IN, SL, and oral (PO), but each has different rates of bioavailability (Table 1) and pharmacokinetics (84, 85). Clinically, these differences may affect efficacy and tolerability. A pharmacokinetic study demonstrated that equal doses of IM, SC, and 40-min IV ketamine infusions achieve similar peak plasma levels and clearance rates (86). Systematic reviews have not demonstrated significantly different side effect or tolerability profiles regardless of the route of administration, though it has been suggested that side effects are most likely correlated with total plasma levels achieved regardless of the route delivered (35, 87).

**Table 1:** Routes and bioavailability of ketamine.

Route	Bioavailability %
Intravenous	95–100
Intramuscular	64–95
Subcutaneous	64–95
Intranasal	30–50
Sublingual	20–30
Oral	10–20

Small studies have demonstrated similar effectiveness/tolerability with IM ketamine at similar doses to IV ketamine in both single and repeat doses (88, 89). A recent RCT (45 patients) also demonstrated equal effectiveness in repeat treatments comparing IM ketamine (0.5 mg/kg), oral ketamine (1 mg/kg), and ECT (90). A systematic review of SC ketamine for depression found safety and efficacy at doses of 0.1–0.5 mg/kg, though studies were noted to be heterogenous in nature (91). A recent RCT (174 patients) investigated SC ketamine, with a more highly treatment-resistant group (more than 5 failed antidepressant trials, 24% failing ECT). Compared to active control (midazolam), doses of 0.6–0.9 mg/kg (but not 0.5 mg/kg) were superior, with a favorable side effect profile (92). Although IM and SC ketamine may offer a more efficient use of resources, saving the time required for IV insertion and infusion, our program has not yet incorporated their use given the comparative lack of studies.

Non-parenteral forms of ketamine including IN, PO and SL ketamine are also reported as safe and efficacious (18). Though less evidence-based than IV ketamine, they may offer improved access due to reduced cost and potentially less monitoring required, which will be later discussed. In our community, expertly-compounded IN or SL racemic ketamine costs $100–150 per month, a significant decrease in treatment cost compared to IN esketamine. While balancing considerations of patient access, safety, and limitations of evidence base for these treatments, concepts have been applied to a paradigm to treat patients with these modalities (83), and physicians in our program have utilized IN and SL ketamine at times for both acute and maintenance treatment. The clinical context of the patient (including degree of illness, previous treatments, treatment setting, resource availability) should be considered in balance with potential side effects/risks. Suggested criteria for offering non parenteral ketamine are highlighted in Table 2.

**Table 2:** Clinical scenarios to consider for non-IV forms of ketamine for acute or maintenance treatment.

Individuals reasonable to consider for an acute course (8 treatments) of non-IV ketamine would be those with both Major depressive episode (unipolar or bipolar), refractory to trials of 2 or more antidepressants/mood stabilizers, and adjunctive agents with a greater evidence base, AND Unable to access more evidence-based ketamine treatments such as IV ketamine and IN esketamine.
Individuals reasonable to consider for maintenance non-IV ketamine treatments would include those with: Major depressive episode (unipolar or bipolar), considered to have exhausted other treatment options with trials of multiple antidepressants, adjuncts, and mood-stabilizing medications from different classes, *but have had positive response to an acute course of either IV or SL/IN ketamine*, AND Unable to access maintenance IV ketamine or IN esketamine treatments. Where patients continue to have coverage and access to IN esketamine following an index series of IN esketamine treatments, we would suggest continuing with IN esketamine for maintenance as it is most strongly supported by the literature at this time in terms of efficacy and safety.

### 3.13 What doses should be used for non-parenteral forms of ketamine?

Though parenteral doses of ketamine in studies have been relatively consistent, evidence for optimal dosing of non-parenteral ketamine remains limited. Though meta-analysis supports safety and efficacy of IN ketamine, most of this data is derived from IN esketamine trials. One report on racemic IN ketamine suggested tolerability concerns (93), but several others support its use. A small, randomized cross-over study found efficacy and favorable tolerability of a single 50 mg dose (94), and a retrospective case series with repeated doses of 100-150 mg noted positive results in the majority of patients with no instances of discontinuation for adverse side effects or concerning safety signals (95). A subsequent retrospective study reported benefit and tolerability for 50 or 75 mg of IN ketamine in psychiatric inpatients with TRD (96). An international consensus paper suggests that compounded racemic IN ketamine could be used in doses ranging from 50 to 150 mg once or twice weekly (29).

Though meta-analysis data is positive for PO and SL ketamine (97), reported doses and frequencies varied widely, ranging from 0.5–1.25 mg/kg (or 50-300 mg for studies which reported total doses only) used multiple times per day to once a month. A recent large (*N* = 664) retrospective report of SL ketamine (300-450 mg) used off-label at home demonstrated nearly identical results to IV ketamine when administered as a series of 6 treatments (98).

As data regarding dosing is limited, we have elected to dose based on bioavailability of the chosen formulation in comparison to IV dosing (100% bioavailable) of 0.5–1.0 mg/kg (Table 3). Though it is a crude estimate that is unable to account for varying pharmacokinetic factors, it has been a useful clinical guideline. If used for acute treatment in our program, IN or SL ketamine is typically given 2–3 times weekly, modeling IV ketamine and IN esketamine schedules. For an initial course to ketamine-naive individuals, patients are started at the minimum effective dose of the calculated dose range.

**Table 3:** Suggested dosing for intranasal and sublingual ketamine.

Mode of administration	Intravenous	Intranasal	Sublingual
Bioavailability (%)	100	30–50	20–30
Minimum effective dose (mg/kg)	0.5	1.0	1.5
Maximal effective dose (mg/kg)	1.0	3.0	5
Conversion/multiplier factor from previously given IV ketamine dose	N/A	2–3	3–5

Some prescribers instead choose not to dose by weight and start ketamine-naïve patients conservatively at 50-100 mg SL or IN, titrating the dose up as tolerated to efficacy. One recent report successfully started ketamine-naïve patients at 300 mg SL and increased as tolerated to 450 mg (98), but our approach to date has been more conservative. A recent chart review of a sample of patients from our program found SL ketamine was generally started at 50-200 mg, though the most common starting doses were 100 mg and 150 mg. Subsequent increases went as high as 300 mg (81). IN ketamine was typically started at 100 mg and increased as high as 150 mg. Starting doses near the higher range would typically be patients transitioned to SL ketamine for maintenance, following a course of IV ketamine. Optimal dosing to maximize the balance between efficacy and tolerability requires further research.

### 3.14 In what setting can patients use SL or in ketamine?

While Health Canada requires IN esketamine to be administered and monitored a health care setting, this mandate is not aligned with the drug’s side effect and risk profile, so should not set a standard SL or IN racemic ketamine use. Significant adverse events have not been reported with esketamine, including issues related to transient hypertension or dissociative side effects. Similarly, long term IN esketamine use has not been associated with safety concerns. Concerns for addiction potential, misuse, or diversion may prompt the tight control around IN esketamine, but it has been previously noted that regulatory policies do not align with expert consensus regarding risks (99). Potential harms of ketamine have been assessed as similar to stimulants or benzodiazepines, all of which are lower than alcohol. Placing ketamine on a more restrictive access and monitoring schedule than other psychotropics with abuse potential stigmatizes this treatment, and limits access for individuals with TRD.

Initially, patients in our program were monitored in office for their first SL or IN treatment, with potential for subsequent treatments to be used at home. However, our critical assessment of risks and benefits concludes that in office monitoring need not be routinely required. As discussed above, blood pressure need not be monitored as asymptomatic hypertension should not be treated. Patients typically report dissociative effects to be less than experienced in with IV ketamine treatments, and with appropriate psychoeducation, dissociative experiences are rarely a concern. Non parenteral forms of ketamine may be safely prescribed for home use, to the appropriate patient, in the appropriate clinical scenario.

Suggested considerations for prescribing ketamine for home use have previously been raised and are summarized in Table 4. If home administration is chosen to be a suitable alternative, other authors have also made practical suggestions for judicious prescribing. These are noted in Table 5. Our program follows these suggestions, and requests patients to return used intranasal devices to the pharmacy for disposal of any remaining ketamine in the device.

**Table 4:** Clinical factors that would support eligibility for less supervised ketamine use.

High level of treatment resistance – patients who have exhausted other treatment options - Severe symptoms - Significant disability - Suicidality - Has required usage of other off-label treatments in the past
No drug misuse history – Substance abuse/misuse screen
No previous history of antisocial/illegal activity/drug diversion
Previous positive response to ketamine
Limited ability to access ketamine treatments with stronger evidence base (IV ketamine or IN esketamine)
Reliable to attend follow-up appointments
Medically suitable for ketamine treatment, including stable cardiovascular status and controlled baseline blood pressure
Compliant with side effect monitoring
Significant experience with side effects of psychotropics and good judgment on reporting these to the clinician

Reprinted with permission from Swainson et al. (83), under license CC BY-NC 4.0.

**Table 5:** Practical suggestions for judicious prescribing of ketamine as an antidepressant for home use.

Obtain and document informed consent – potential risks/benefits
Consider the use of patient contracts
Prescribe in limited quantities and limited refills (for example, provide 2–4 weeks supply depending on frequency of dosing)
Prescriber experience with ketamine - Affiliation with a more intensive ketamine program (for further assessment/referral/case discussions)
Consider observing first treatments or dose changes in office to monitor blood pressure in medically at-risk patients, and dissociation in ketamine-naïve individuals
Educate patients on dissociative symptoms
Advise patients not to drive until next day after use Dose at night when used at home Advise a quiet calm environment For ketamine-naïve patients, consider the presence of another responsible adult for the first several doses, if not observed in office Wait until dissociative/sedative effects of ketamine dissipate before using other potentially sedating bedtime medications
Screen for bladder toxicity - Check urinalysis at baseline and q3-6 months for signs of microscopic hematuria - Ask about urinary symptoms (for example, frequency, urgency, hematuria)
Monitor for drug liking/signs or symptoms of misuse - lost prescriptions - requests for early refills - requests for dose escalation or increased frequency despite stable psychiatric status
Consider that non-IV forms may require higher doses due to reduced bioavailability, and that documented bioavailability of each formulation is to be considered a rough estimate and may vary
Prescribing clinicians should be informed of current literature and continue medical education on ketamine to learn and adjust prescribing practices as new data become available

Adapted with permission from Swainson et al. (83), under license CC BY-NC 4.0.

## 4 Future directions

Future directions in ketamine treatment could include consideration of 3 treatment models we refer to as: (1) step-down approach, (2) step-up approach, and (3) clinical-matching approach. The common aim of all is to find a complementary way to integrate use of SL or IN ketamine into the IV ketamine treatment paradigm. Our program organically evolved into using the “step-down” approach where a course of IV ketamine is administered for acute treatment, and if maintenance treatment is needed, “stepping down” to IN or SL ketamine. This has been done either with a direct transition from an acute course of IV ketamine to SL or IN ketamine at varying intervals or has been done to support an IV ketamine taper, continuing SL or IN treatments once or twice weekly, in between biweekly, every 3 week and then monthly IV treatments, with an eventual goal to transition off IV treatments entirely. While there is no data to support superiority for either mode, a recent report of transition to IN esketamine to maintain the response of IV ketamine provides comparative support for a step-down model (100).

The “step-up” approach is an alternative that could be considered, particularly by new ketamine programs. In this model, a referral criterion for IV ketamine could include a previous failed trial of SL or IN ketamine in the community. This model also evolved organically for during the COVID-19 pandemic, during a time that our IV programs were not operating, and patients were reluctant to come for treatment in a health care setting. SL or IN compounded ketamine was prescribed to ketamine naïve patients with several patients responding well and not requiring IV treatment. This approach could reduce wait lists and reserve IV ketamine treatments for more treatment-resistant individuals. Adopting this “step-up” paradigm could be of particular use in smaller centers with limited IV ketamine availability.

A third “clinical-matching” model would involve making decisions regarding what form of ketamine is appropriate based on patient profile. The patients with TRD that meet criteria for IV ketamine are a heterogeneous group in terms of symptom severity and chronicity, comorbid conditions, and the number of previous treatments tried. While this model has not been used to date in our program, theoretically, IV ketamine could be reserved for only the most treatment resistant patients, such as our original URD population. Other patients who meet “minimum” TRD criteria may respond favorably to SL or IN ketamine treatment. While our clinical experience supports the notion that the “less” treatment resistant patient may respond to SL or IN ketamine alone, further research is needed in this area.

Continued challenges with any of these paradigms include lack of data regarding optimal dosing and frequency for SL and IN ketamine. A clear limitation in our approach is the extrapolation of IV ketamine and IN esketamine data. A review of clinicaltrials.gov indicates that several studies involving racemic IN ketamine are in various stages of progress, so further data to guide its use may be on the horizon. Larger controlled trials with IN/SL ketamine, and comparative studies with IV ketamine or IN esketamine would be of great value to guide future treatment.

## 5 Conclusion

As the evidence for IV ketamine and IN esketamine for TRD has increased, the availability and accessibility of these treatments has been a financial and logistical challenge for many, preventing access to evidence-based treatments with much promise. This community case study has described the evolution of a public ketamine program, including the application of a recent literature review to clinical programming. Sites starting an IV ketamine program must be aware of limitations, particularly in consideration for how maintenance treatment may be offered to those who require it. Without the ability to offer maintenance ketamine in some form, offering this treatment to patients who may respond well, only to relapse again raises questions surrounding the ethics of offering short term treatment only. As we have shared in this community case study, the use of SL and IN ketamine in Edmonton, Canada has facilitated increased access to ketamine treatment, and allowed us to address this issue.

Though awareness of potential risks of ketamine use is essential, it need not be stigmatized as an overly dangerous treatment when considering highly ill and treatment-resistant patients. Rare, but serious adverse events can occur with any treatment, and there is no suggestion that the risks of ketamine are out of keeping with other medications commonly used in psychiatry. The ability to prescribe SL and IN ketamine provides psychiatrists with more options to offer to patients with TRD. Clinicians who elect to offer these treatments must be aware of the limitations in the guiding body of literature. Aspects such as patient selection, regular follow up, and ongoing assessment of risk/benefit for the individual patient are essential. Future research to better elucidate optimal prescribing of ketamine will support physicians and patients in making treatment decisions.

## Data availability statement

The original contributions presented in the study are included in the article/supplementary material, further inquiries can be directed to the corresponding authors.

## Ethics statement

Ethical approval was not required for the studies involving humans because this paper contains no original data. It reviews previously reported research on human populations. The studies were conducted in accordance with the local legislation and institutional requirements. Written informed consent for participation was not required from the participants or the participants’ legal guardians/next of kin in accordance with the national legislation and institutional requirements because there were no participants. Data presented has been reported elsewhere and is reviewed.

## Author contributions

CC: Conceptualization, Methodology, Writing – original draft, Writing – review & editing, Data curation, Resources, Software, Visualization. BD: Conceptualization, Data curation, Visualization, Writing – review & editing. AK: Conceptualization, Visualization, Writing – review & editing. CM: Conceptualization, Writing – review & editing. RT: Conceptualization, Writing – review & editing. JS: Conceptualization, Writing – review & editing, Methodology, Project administration, Supervision, Writing – original draft.
